# Redetermination of *cis*-diaqua­diglycolato­zinc(II)

**DOI:** 10.1107/S1600536808039585

**Published:** 2008-11-29

**Authors:** Paul Kennedy, Neferterneken Francis, David Rovnyak, Margaret E. Kastner

**Affiliations:** a Department of Chemistry, Bucknell University, Lewisburg, PA 17837, USA

## Abstract

The title complex, [Zn(C_2_H_3_O_3_)_2_(H_2_O)_2_], was prepared and the crystal structure determined as part of a ^67^Zn solid state nuclear magnetic resonance study. In the title complex, the Zn atom has a disorted octa­hedral coordination comprising two bidentate glycolate ligands and two water mol­ecules. The water mol­ecules are *cis* to each other; one is *trans* to a carboxyl­ate O atom and the other *trans* to an alcohol O atom. The crystal structure has an extensive O—H⋯O hydrogen-bond network.

## Related literature

The crystal structure of the title complex was first reported by Fischinger & Webb (1969 ▶). For bond-length data, see: Allen *et al.* (1987 ▶).
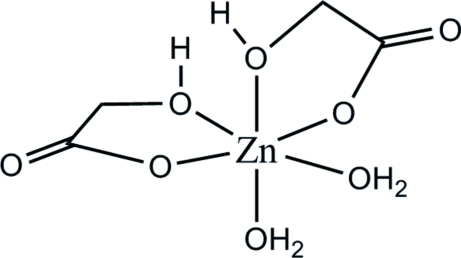


## Experimental

### 

#### Crystal data

[Zn(C_2_H_3_O_3_)_2_(H_2_O)_2_]
*M* *_r_* = 251.49
Monoclinic, 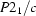

*a* = 11.391 (2) Å
*b* = 5.857 (1) Å
*c* = 12.511 (2) Å
β = 91.198 (9)°
*V* = 834.5 (2) Å^3^
*Z* = 4
Mo *K*α radiation
μ = 2.96 mm^−1^
*T* = 273 (2) K
0.3 × 0.3 × 0.1 mm

#### Data collection

Bruker P4 diffractometer
Absorption correction: ψ scan (*SADABS*; Bruker, 2000 ▶) *T*_min_ = 0.425, *T*_max_ = 0.744
2102 measured reflections
2102 independent reflections
1751 reflections with *I* > 2σ(*I*)
3 standard reflections every 97 reflections intensity decay: none

#### Refinement

*R*[*F* ^2^ > 2σ(*F* ^2^)] = 0.030
*wR*(*F* ^2^) = 0.072
*S* = 0.99
2102 reflections
119 parameters
H-atom parameters constrained
Δρ_max_ = 0.45 e Å^−3^
Δρ_min_ = −0.51 e Å^−3^

### 

Data collection: *XSCANS* (Bruker, 2000 ▶); cell refinement: *XSCANS*; data reduction: *XSCANS*; program(s) used to solve structure: *SHELXS97* (Sheldrick, 2008 ▶); program(s) used to refine structure: *SHELXL97* (Sheldrick, 2008 ▶); molecular graphics: *SHELXTL* (Sheldrick, 2008 ▶); software used to prepare material for publication: *SHELXTL*.

## Supplementary Material

Crystal structure: contains datablocks I, global. DOI: 10.1107/S1600536808039585/su2079sup1.cif

Structure factors: contains datablocks I. DOI: 10.1107/S1600536808039585/su2079Isup2.hkl

Additional supplementary materials:  crystallographic information; 3D view; checkCIF report

## Figures and Tables

**Table 1:** Hydrogen-bond geometry (Å, °)

*D*—H⋯*A*	*D*—H	H⋯*A*	*D*⋯*A*	*D*—H⋯*A*
O1—H8⋯O4^i^	0.90	1.93	2.821 (3)	167
O1—H7⋯O8^ii^	0.88	1.84	2.716 (3)	174
O2—H6⋯O5^i^	0.91	1.82	2.697 (3)	161
O2—H5⋯O5^iii^	0.85	1.88	2.688 (3)	158
O6—H4⋯O8^iv^	0.84	1.82	2.665 (2)	177
O3—H3⋯O7^iv^	0.83	1.97	2.761 (3)	160

Symmetry codes: (i) 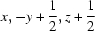; (ii) 
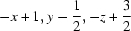
; (iii) 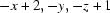; (iv) 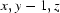.

**Fig. 1.**
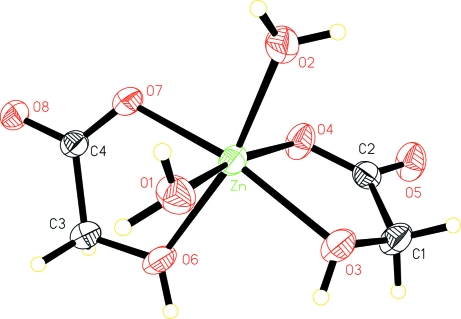
A view of the molecular structure of compound (I), showing the atom numbering scheme and dispacement ellipsods drawn at the 50% probability level.

## supplementary crystallographic information

### Comment

As part of a Zinc-67 solid state nuclear magnetic resonance study the title complex, (I), was prepared and the crystal structure determined. The structure of this complex was first report by Fischinger & Webb (1969) but no fractional crystal coordinates were reported.

The molecular structure of (I) is illustrated in Fig. 1. The zinc atom has a distorted octahedral coordination sphere composed of two bidentate gylcolato ligands and two water molecules. The water molecules are *cis* to each other; one (O1) is *trans* to a carboxylate O-atom (O4), and the other, (O2), is *trans* to an alcohol O-atom (O6). The bond distances and angles are normal for zinc(II) complexes (Allen *et al.*, 1987)

In the crystal structure of (I) there in an extensive O—H···O hydrogen bonding network (Table 1). The two water molecules (O1 and O2) bond to the two oxygens (O4 and O5) of a carboxylate group related by the c-glide. The two alcohol groups (O3 and O6) form hydrogen bonds with the other carboxylate group (atoms O7 and O8) translated by one unit cell along the b axis. The carbonyl oxygen O8 of this ligand also makes a two dimensional hydrogen bonded network with one of the waters (O1) around the inversion center. The other water molecule, (O2), forms an H-bond with a carbonyl oxygen (O5) related by the 2-fold screw axis.

### Experimental

Glycolic acid (100 mg, 3 mmol), purchased from Sigma-Aldrich (99%), was dissolved in 5 ml of deionized water. Basic zinc carbonate (80 mg, 2 mmol) was added and the mixture stirred for 10 minutes while heating to ca. 60°C. The resulting mixture was filtered, and the filtrate left to stand at room temperature until large needle-like crystals grew by slow evaporation of the water.

### Refinement

The alcohol and water H-atoms were placed at the locations identified in a difference Fourier map and were held fixed, with U_iso_(H) set to 0.05 A^2^: O-H = 0.8293 - 0.9135 Å. The C-bound H-atoms were included in calculated positions and treated as riding atoms: C-H = 0.97 Å with U_iso_(H) = 1.2U_eq_(parent C-atom).

### Crystal data

**Table:**

[Zn(C_2_H_3_O_3_)_2_(H_2_O)_2_]	*F*_000_ = 512
*M**_r_* = 251.49	*D*_x_ = 2.002 Mg m^−^^3^
Monoclinic, *P*2_1_/*c*	Mo *K*α radiation λ = 0.71073 Å
Hall symbol: -P 2ybc	Cell parameters from 20 reflections
*a* = 11.391 (2) Å	θ = 9.6–17.4º
*b* = 5.8570 (10) Å	µ = 2.96 mm^−^^1^
*c* = 12.511 (2) Å	*T* = 273 (2) K
β = 91.198 (9)º	Needle, colorless
*V* = 834.5 (2) Å^3^	0.3 × 0.3 × 0.1 mm
*Z* = 4	

### Data collection

**Table:**

Bruker P4 diffractometer	*R*_int_ = 0.0000
Radiation source: fine-focus sealed tube	θ_max_ = 28.5º
Monochromator: graphite	θ_min_ = 3.3º
*T* = 273(2) K	*h* = −15→15
2θ/ω scans	*k* = 0→7
Absorption correction: ψ scan(SADABS; Bruker, 2000)	*l* = 0→16
*T*_min_ = 0.425, *T*_max_ = 0.744	3 standard reflections
2102 measured reflections	every 97 reflections
2102 independent reflections	intensity decay: none
1751 reflections with *I* > 2σ(*I*)	

### Refinement

**Table:**

Refinement on *F*^2^	Hydrogen site location: inferred from neighbouring sites
Least-squares matrix: full	H-atom parameters constrained
*R*[*F*^2^ > 2σ(*F*^2^)] = 0.030	*w* = 1/[σ^2^(*F*_o_^2^) + (0.0337*P*)^2^ + 0.5879*P*] where *P* = (*F*_o_^2^ + 2*F*_c_^2^)/3
*wR*(*F*^2^) = 0.072	(Δ/σ)_max_ = 0.030
*S* = 0.99	Δρ_max_ = 0.45 e Å^−^^3^
2102 reflections	Δρ_min_ = −0.50 e Å^−^^3^
119 parameters	Extinction correction: SHELXL97 (Sheldrick, 2008), Fc^*^=kFc[1+0.001xFc^2^λ^3^/sin(2θ)]^-1/4^
Primary atom site location: structure-invariant direct methods	Extinction coefficient: 0.0094 (8)
Secondary atom site location: difference Fourier map	

### Special details

**Table:**

Geometry. All e.s.d.'s (except the e.s.d. in the dihedral angle between two l.s. planes) are estimated using the full covariance matrix. The cell e.s.d.'s are taken into account individually in the estimation of e.s.d.'s in distances, angles and torsion angles; correlations between e.s.d.'s in cell parameters are only used when they are defined by crystal symmetry. An approximate (isotropic) treatment of cell e.s.d.'s is used for estimating e.s.d.'s involving l.s. planes.
Refinement. Refinement of *F*^2^ against ALL reflections. The weighted *R*-factor *wR* andgoodness of fit *S* are based on *F*^2^, conventional *R*-factors *R* are basedon *F*, with *F* set to zero for negative *F*^2^. The threshold expression of*F*^2^ > σ(*F*^2^) is used only for calculating *R*-factors(gt) *etc*. and isnot relevant to the choice of reflections for refinement. *R*-factors basedon *F*^2^ are statistically about twice as large as those based on *F*, and *R*-factors based on ALL data will be even larger.

### Fractional atomic coordinates and isotropic or equivalent isotropic displacement parameters (Å^2^)

**Table:**

	*x*	*y*	*z*	*U*_iso_*/*U*_eq_	
Zn	0.76227 (2)	0.14144 (5)	0.65837 (2)	0.02431 (11)	
O1	0.73409 (15)	0.0926 (3)	0.82160 (13)	0.0323 (4)	
O2	0.91409 (16)	0.3091 (3)	0.69490 (15)	0.0349 (4)	
O3	0.82248 (17)	−0.1945 (3)	0.64048 (13)	0.0307 (4)	
O4	0.80776 (17)	0.1349 (3)	0.49645 (13)	0.0310 (4)	
O5	0.90455 (17)	−0.0608 (4)	0.37503 (13)	0.0367 (4)	
O6	0.58388 (16)	0.0302 (3)	0.63177 (15)	0.0339 (4)	
O7	0.66840 (15)	0.4419 (3)	0.64627 (14)	0.0299 (4)	
O8	0.49499 (15)	0.6096 (3)	0.62300 (13)	0.0275 (4)	
C1	0.8665 (2)	−0.2479 (4)	0.53732 (19)	0.0297 (5)	
H1A	0.8215	−0.3727	0.5062	0.036*	
H1B	0.9477	−0.2968	0.5444	0.036*	
C2	0.8588 (2)	−0.0423 (4)	0.46432 (18)	0.0242 (5)	
C3	0.4989 (2)	0.2052 (4)	0.62244 (18)	0.0242 (5)	
H2A	0.4422	0.1896	0.6787	0.029*	
H2B	0.4575	0.1927	0.5542	0.029*	
C4	0.5587 (2)	0.4364 (4)	0.63092 (16)	0.0218 (4)	
H3	0.7697	−0.2865	0.6547	0.050*	
H4	0.5587	−0.1048	0.6293	0.050*	
H5	0.9803	0.2654	0.6719	0.050*	
H6	0.9284	0.3898	0.7563	0.050*	
H7	0.6612	0.0892	0.8427	0.050*	
H8	0.7688	0.1759	0.8742	0.050*	

### Atomic displacement parameters (Å^2^)

**Table:**

	*U*^11^	*U*^22^	*U*^33^	*U*^12^	*U*^13^	*U*^23^
Zn	0.02730 (16)	0.01890 (15)	0.02686 (15)	0.00221 (12)	0.00378 (10)	−0.00151 (11)
O1	0.0314 (9)	0.0381 (10)	0.0275 (8)	−0.0050 (8)	0.0044 (7)	−0.0032 (7)
O2	0.0280 (9)	0.0360 (10)	0.0408 (10)	−0.0002 (8)	0.0038 (7)	−0.0114 (8)
O3	0.0452 (10)	0.0204 (8)	0.0269 (8)	0.0026 (8)	0.0121 (7)	0.0011 (7)
O4	0.0404 (10)	0.0270 (9)	0.0260 (8)	0.0094 (8)	0.0063 (7)	0.0037 (7)
O5	0.0435 (11)	0.0390 (11)	0.0280 (9)	0.0100 (9)	0.0116 (8)	0.0036 (8)
O6	0.0327 (10)	0.0161 (8)	0.0527 (11)	0.0002 (8)	−0.0013 (8)	−0.0041 (8)
O7	0.0268 (9)	0.0174 (8)	0.0457 (10)	0.0012 (7)	0.0026 (7)	−0.0014 (8)
O8	0.0294 (9)	0.0198 (8)	0.0333 (9)	0.0036 (7)	0.0046 (7)	0.0029 (7)
C1	0.0414 (14)	0.0203 (11)	0.0277 (12)	0.0026 (11)	0.0100 (10)	−0.0006 (10)
C2	0.0216 (11)	0.0255 (12)	0.0257 (11)	−0.0017 (10)	0.0017 (8)	0.0014 (9)
C3	0.0294 (12)	0.0196 (10)	0.0238 (10)	−0.0008 (10)	−0.0008 (9)	−0.0002 (9)
C4	0.0284 (11)	0.0185 (10)	0.0188 (9)	−0.0011 (9)	0.0049 (8)	0.0010 (8)

### Geometric parameters (Å, °)

**Table:**

Zn—O2	2.0325 (19)	O4—C2	1.259 (3)
Zn—O7	2.0630 (17)	O5—C2	1.247 (3)
Zn—O1	2.0935 (17)	O6—C3	1.412 (3)
Zn—O3	2.0974 (18)	O6—H4	0.8417
Zn—O4	2.1019 (17)	O7—C4	1.261 (3)
Zn—O6	2.1531 (19)	O8—C4	1.250 (3)
O1—H7	0.8768	C1—C2	1.513 (3)
O1—H8	0.9037	C1—H1A	0.9700
O2—H5	0.8522	C1—H1B	0.9700
O2—H6	0.9135	C3—C4	1.518 (3)
O3—C1	1.429 (3)	C3—H2A	0.9700
O3—H3	0.8293	C3—H2B	0.9700
			
O2—Zn—O7	92.38 (7)	C2—O4—Zn	116.58 (15)
O2—Zn—O1	89.65 (7)	C3—O6—Zn	115.85 (14)
O7—Zn—O1	95.65 (7)	C3—O6—H4	116.5
O2—Zn—O3	101.47 (8)	Zn—O6—H4	127.6
O7—Zn—O3	164.27 (7)	C4—O7—Zn	120.00 (16)
O1—Zn—O3	91.89 (7)	O3—C1—C2	110.72 (19)
O2—Zn—O4	89.99 (7)	O3—C1—H1A	109.5
O7—Zn—O4	94.74 (7)	C2—C1—H1A	109.5
O1—Zn—O4	169.61 (7)	O3—C1—H1B	109.5
O3—Zn—O4	78.01 (7)	C2—C1—H1B	109.5
O2—Zn—O6	167.62 (7)	H1A—C1—H1B	108.1
O7—Zn—O6	76.15 (7)	O5—C2—O4	124.2 (2)
O1—Zn—O6	86.90 (7)	O5—C2—C1	116.8 (2)
O3—Zn—O6	90.53 (7)	O4—C2—C1	119.00 (19)
O4—Zn—O6	95.53 (7)	O6—C3—C4	109.64 (19)
Zn—O1—H7	117.5	O6—C3—H2A	109.7
Zn—O1—H8	124.3	C4—C3—H2A	109.7
H7—O1—H8	101.3	O6—C3—H2B	109.7
Zn—O2—H5	122.2	C4—C3—H2B	109.7
Zn—O2—H6	125.1	H2A—C3—H2B	108.2
H5—O2—H6	107.2	O8—C4—O7	124.3 (2)
C1—O3—Zn	115.05 (14)	O8—C4—C3	117.4 (2)
C1—O3—H3	108.8	O7—C4—C3	118.3 (2)
Zn—O3—H3	110.3		
			
O2—Zn—O3—C1	−84.29 (19)	O2—Zn—O7—C4	−174.79 (18)
O7—Zn—O3—C1	67.0 (3)	O1—Zn—O7—C4	−84.91 (18)
O1—Zn—O3—C1	−174.31 (18)	O3—Zn—O7—C4	33.4 (4)
O4—Zn—O3—C1	3.24 (18)	O4—Zn—O7—C4	95.01 (18)
O6—Zn—O3—C1	98.77 (18)	O6—Zn—O7—C4	0.47 (17)
O2—Zn—O4—C2	94.74 (19)	Zn—O3—C1—C2	0.1 (3)
O7—Zn—O4—C2	−172.86 (19)	Zn—O4—C2—O5	−170.4 (2)
O1—Zn—O4—C2	6.7 (5)	Zn—O4—C2—C1	9.4 (3)
O3—Zn—O4—C2	−6.98 (18)	O3—C1—C2—O5	173.5 (2)
O6—Zn—O4—C2	−96.36 (19)	O3—C1—C2—O4	−6.3 (3)
O2—Zn—O6—C3	21.4 (5)	Zn—O6—C3—C4	1.6 (2)
O7—Zn—O6—C3	−1.22 (16)	Zn—O7—C4—O8	179.73 (17)
O1—Zn—O6—C3	95.41 (17)	Zn—O7—C4—C3	0.3 (3)
O3—Zn—O6—C3	−172.75 (16)	O6—C3—C4—O8	179.2 (2)
O4—Zn—O6—C3	−94.73 (17)	O6—C3—C4—O7	−1.3 (3)

### Hydrogen-bond geometry (Å, °)

**Table:**

*D*—H···*A*	*D*—H	H···*A*	*D*···*A*	*D*—H···*A*
O1—H8···O4^i^	0.90	1.93	2.821 (3)	167
O1—H7···O8^ii^	0.88	1.84	2.716 (3)	174
O2—H6···O5^i^	0.91	1.82	2.697 (3)	161
O2—H5···O5^iii^	0.85	1.88	2.688 (3)	158
O6—H4···O8^iv^	0.84	1.82	2.665 (2)	177
O3—H3···O7^iv^	0.83	1.97	2.761 (3)	160

Symmetry codes: (i) *x*, −*y*+1/2, *z*+1/2; (ii) −*x*+1, *y*−1/2, −*z*+3/2; (iii) −*x*+2, −*y*, −*z*+1; (iv) *x*, *y*−1, *z*.
